# Statement of Retraction: Upregulation of long non-coding RNA NNT-AS1 promotes osteosarcoma progression by inhibiting the tumor suppressive miR-320a

**DOI:** 10.1080/15384047.2026.2740298

**Published:** 2026-09-29

We, the Editors and Publisher of *Cancer Biology & Therapy*, have retracted the following article:

Li, C., Zhang, S., Qiu, T., Wang, Y., Ricketts, D. M., & Qi, C. (2019). Upregulation of long non-coding RNA NNT-AS1 promotes osteosarcoma progression by inhibiting the tumor suppressive miR-320a. *Cancer Biology & Therapy*, *20*(4), 413–422. https://doi.org/10.1080/15384047.2018.1538612

Following publication, concerns were raised by a third party regarding the image integrity of Transwell assay Figures 1 G, 4I, Colony formation assay 1 C, Tumour formation assay 5D, and Scratch assay Figure 1I.

Further investigation by the Publisher additionally identified image integrity concerns with Flow Cytometry Figures 1E, 4 G, Western Blot Figures 2 C, 2 H, 3E, 4D and the qRT-PCR methodology and results described in the article. When approached for an explanation, the authors did not respond to our queries.

As verifying the validity of published work is core to the integrity of the scholarly record, we are therefore retracting the article. The corresponding author has been informed.

We have been informed in our decision-making by our editorial policies and the COPE guidelines.

The retracted article will remain online to maintain the scholarly record, but it will be digitally watermarked on each page as ‘Retracted’.

